# Adrenocortical Carcinoma in Peutz–Jeghers Syndrome With a Rare *STK11* Pathogenic Germline Variant: A Case Report

**DOI:** 10.1002/cnr2.70583

**Published:** 2026-05-24

**Authors:** Tomoki Ishida, Shuya Kandori, Reo Takahashi, Satoshi Nitta, Masanobu Shiga, Yoshiyuki Nagumo, Takashi Kawahara, Akio Hoshi, Hiromitsu Negoro, Bryan J. Mathis, Shohei Sugita, Daisuke Matsubara, Toshitaka Ishiguro, Takahito Nakajima, Hiroyuki Nishiyama

**Affiliations:** ^1^ Department of Urology University of Tsukuba Hospital Tsukuba Ibaraki Japan; ^2^ Department of Urology, Institute of Medicine University of Tsukuba Tsukuba Ibaraki Japan; ^3^ Department of Cardiovascular Surgery, Institute of Medicine University of Tsukuba Tsukuba Ibaraki Japan; ^4^ Department of Pathology University of Tsukuba Hospital Tsukuba Ibaraki Japan; ^5^ Department of Pathology, Institute of Medicine University of Tsukuba Tsukuba Ibaraki Japan; ^6^ Department of Radiology University of Tsukuba Hospital Tsukuba Ibaraki Japan; ^7^ Department of Radiology, Institute of Medicine University of Tsukuba Tsukuba Ibaraki Japan

**Keywords:** adrenocortical carcinoma, case report, Peutz–Jeghers syndrome, *STK11*

## Abstract

**Background:**

Peutz–Jeghers syndrome (PJS) is an inherited, autosomal‐dominant condition, featuring *STK11* germline mutations, characterized by hamartomatous gastrointestinal polyps and increased cancer risk. The most commonly associated malignancies are gastrointestinal, pancreatic, and breast cancers. Herein, we report an extremely rare case of PJS with adrenocortical carcinoma (ACC).

**Case:**

A 40‐year‐old woman was referred to University of Tsukuba Hospital with uncontrolled diabetes mellitus (DM) and hypokalemia in July 2021. Computed tomography (CT) and magnetic resonance imaging (MRI) revealed a 12 cm mass in the right adrenal gland and aortocaval lymph node swelling. Mucocutaneous hyperpigmentation in her lips and fingertips was observed, and multiple stomach and small intestine polyps were visible on CT. We performed open right adrenalectomy with right nephrectomy, partial hepatectomy, and aortocaval lymph node dissection before diagnosing ACC with lymph node metastasis from the pathology. After surgery, multiple lung and liver metastases developed, and combination chemotherapy (etoposide, doxorubicin, and cisplatin, plus mitotane) was started, achieving stable disease for 2 years. We also performed an OncoGuide NCC Oncopanel on the adrenal tumor specimen and blood sample, revealing a rarely reported pathogenic germline variant (c.394T>C, p.C132R) in *STK11*. This variant was registered as Likely Pathogenic in the NIH Clinvar public database, which led to a PJS diagnosis.

**Conclusion:**

This is the second report of ACC associated PJS in a rare germline variant of *STK11*. Although rare, loss of *STK11* function may lead to cancers outside expected sites in PJS cases.

**Abbreviations**
ACC: Adrenocortical carcinoma
*AMPK*
: AMP activated protein kinase
CT: computed tomography
DHEA‐S: dehydroepiandrosterone sulfate
DM: diabetes
EDP‐M: etoposide, doxorubicin and cisplatin plus mitotane
*LKB1*
: liver kinase B1
MRI: magnetic resonance imaging
MSI: microsatellite instability
PD: progressive disease
PJS: Peutz–Jeghers syndrome
SD: stable disease
*STK11*
: serine/threonine kinase 11
TMB: tumor mutation burden

## 1 Introduction

Peutz–Jeghers syndrome (PJS) is autosomal‐dominant and features gastrointestinal, hamartomatous polyposis along with characteristic mucocutaneous hyperpigmentation [1] caused by a pathological germline variant of the serine/threonine kinase 11 (*STK11*) gene. It imparts a significant increase in epithelial malignancy risk, especially gastrointestinal malignancies (10%–40%) and breast cancer (54%) [1]. However, the incidence of adrenocortical carcinoma (ACC) in patients with PJS is extremely rare [2].

Here, we report such a case of PJS presenting with ACC, confirming a similarly rare pathogenic germline *STK11* mutation (c.394T>C, p.C132R).

## 2 Case Report

**FIGURE 1:**
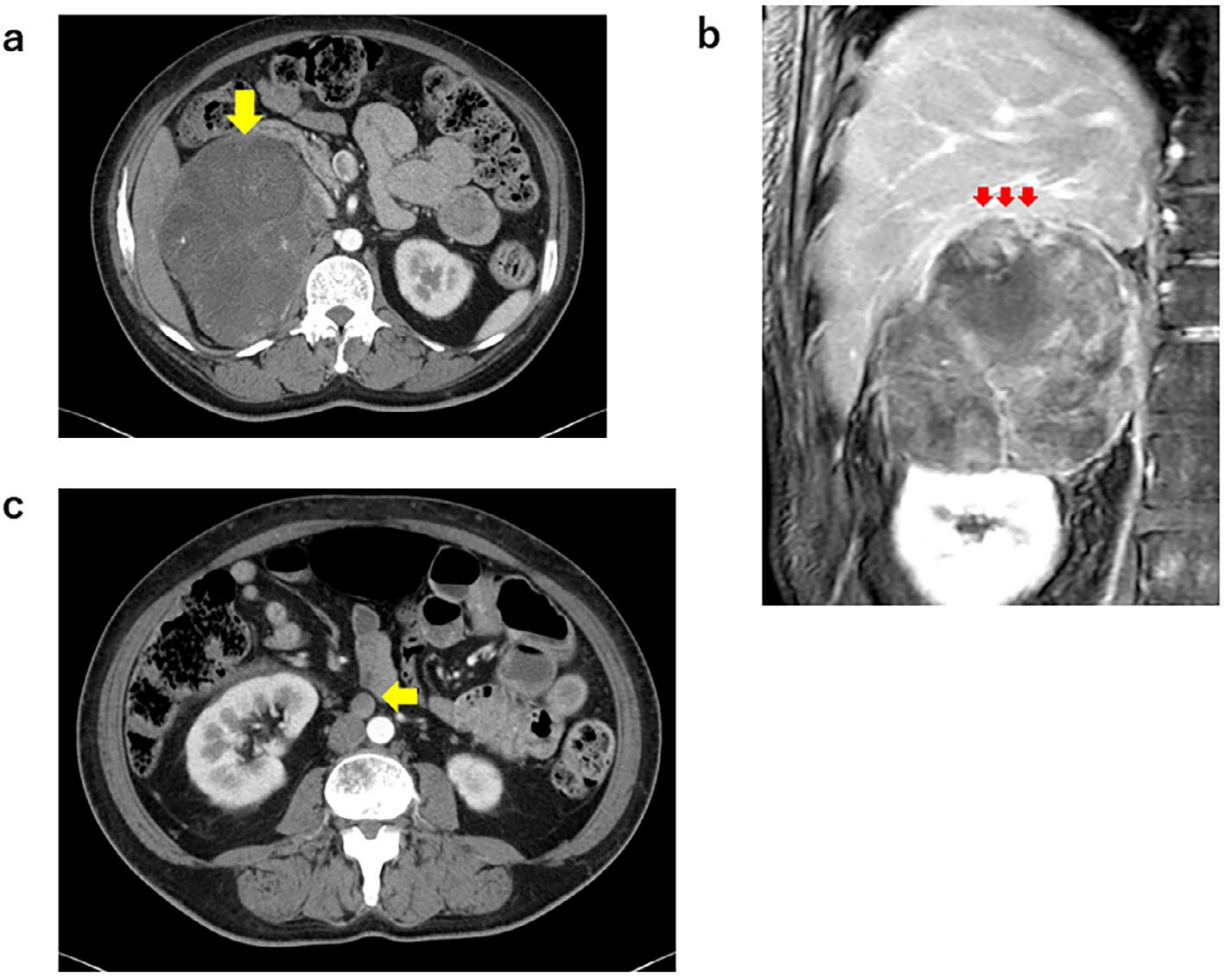
Imaging findings of a right adrenal tumor. (a) Enhanced CT finding of a right adrenal tumor. (b) Enhanced MR finding of a right adrenal tumor. Arrow indicates a suspected liver invasion. (c) Enhanced CT finding of a right adrenal tumor and a paracaval lymph node metastasis (indicated by arrow).

**FIGURE 2:**
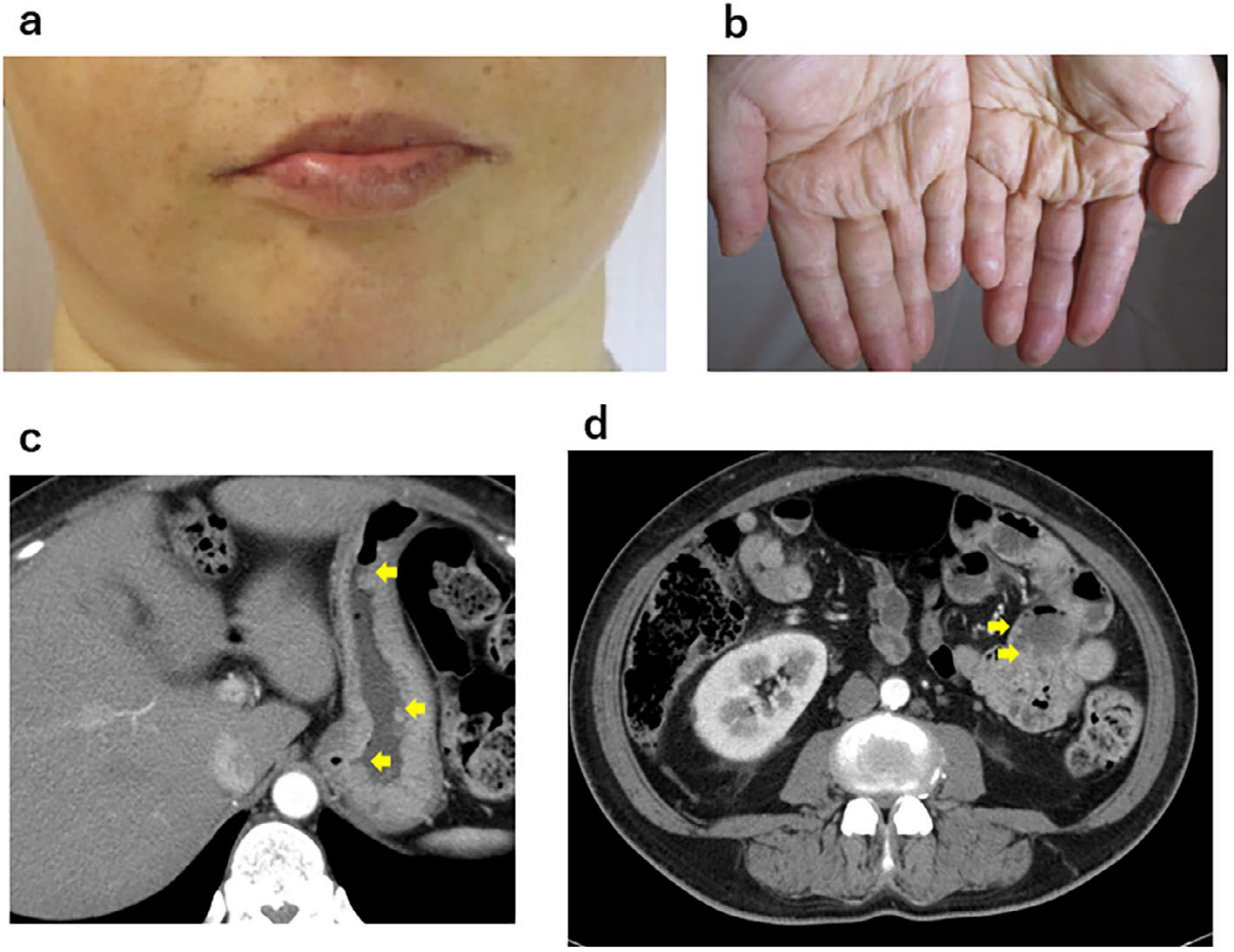
Findings pointing to Peutz–Jeghers syndrome. (a, b) Mucocutaneous pigmentation on the lips (a) and fingers (b). (c, d) CT finding of multiple gastric polyps (c) and small intestine polyps (d). Arrows indicate polyps.

A 40‐year‐old woman was referred to University of Tsukuba Hospital with uncontrolled diabetes mellitus (DM) and hypokalemia in July 2021. Physical examination revealed hirsutism, signs of masculinization, and marked edema of the lower legs. Serum cortisol (17.1 μg/dL, reference range: 6.4–21.0 μg/dL) and testosterone (1.44 ng/mL, reference range: 0.8–1.7 ng/mL) levels were within normal limits, whereas serum aldosterone (145 pg/mL, reference range: 4.0–82.1 pg/mL) and dehydroepiandrosterone sulfate (DHEA‐S) (1100 μg/dL, reference range: 19–231 μg/dL) levels were elevated. Computed tomography (CT) and magnetic resonance imaging (MRI) revealed a 12 cm large mass in the right adrenal gland that had extensively invaded the renal, liver, and anterior renal fascia (Figure 1a,b). Aortocaval lymph node metastasis was also suspected (Figure 1c). Coincidentally, our radiologist found mucocutaneous hyperpigmentation in her lips and fingertips during the imaging study (Figure 2a,b), and CT findings revealed multiple polyps in her stomach and small intestine (Figure 2c). These results collectively indicated PJS but relevant history was unavailable due to the death of family members during childhood.

**FIGURE 3:**
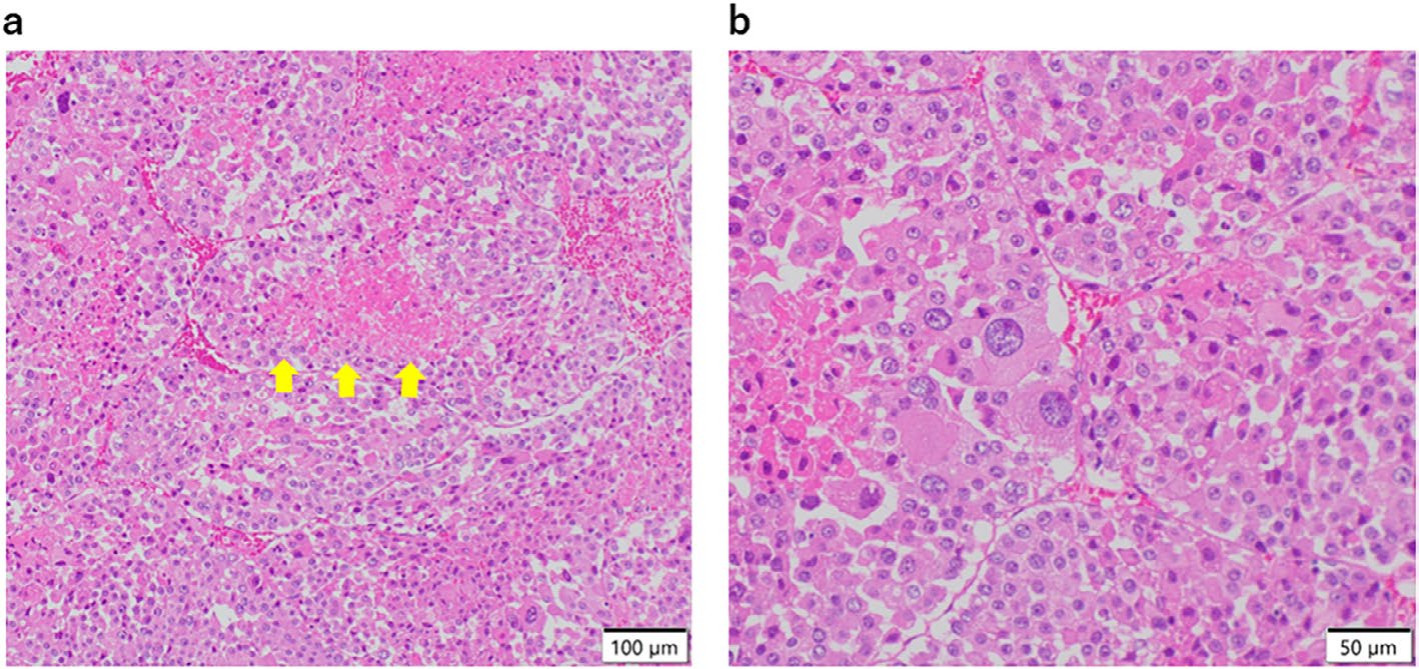
Pathological findings of a right adrenal tumor. (a, b) HE staining at low magnification (×100) (a) and high magnification (×200) (b). Arrows indicate tumor necrosis.

An open right adrenalectomy, right nephrectomy, partial hepatectomy and aortocaval lymph node dissection were performed. Histologic examination revealed eosinophilic tumor cells which had partially invaded into the adipose tissue (Figure 3a,b). Tumor necrosis was found (Figure 3a) and the mitotic activity was 15/50 HPF (Figure 3b), with a Weiss score of 6 points, leading to a diagnosis of ACC with lymph node metastasis (pT3N1M0, StageIII).

**FIGURE 4:**
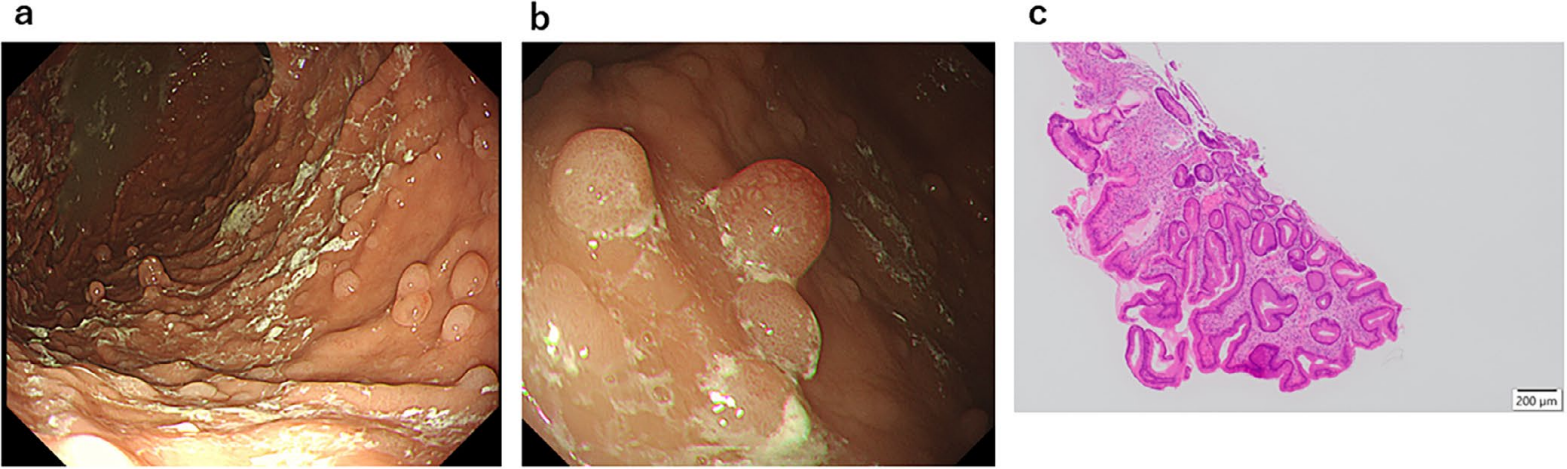
Finding of multiple gastrointestinal polyps. (a) Stomach endoscopy. (b) Duodenal endoscopy. (c) Pathological findings of a gastric polyp at low magnification.

A month after surgery, multiple lung metastases were spotted on CT and mitotane monotherapy was started because she first refused chemotherapy. However, since the lung metastases progressed and a liver metastasis newly appeared, combination chemotherapy with etoposide, doxorubicin, and cisplatin plus mitotane (EDP‐M) was started. We administered doxorubicin (40 mg/m^2^) on Day 1; etoposide (75 mg/m^2^) on Days 2, 3, and 4; and cisplatin (30 mg/m^2^) on Days 3 and 4, every 28 days. The dose and administration schedule for EDP‐M therapy follow the guidelines established in the FIRM‐ACT trial and European Society of Endocrinology Clinical Practice Guidelines [3, 4]. Etoposide and cisplatin were administered at 75% of the standard dose due to renal dysfunction (creatinine clearance 47 mL/min). We also performed an OncoGuide NCC Oncopanel as a cancer gene panel test with the adrenal tumor specimen and blood sample. High tumor mutation burden (TMB‐H), high microsatellite instability (MSI‐H), and pathogenic somatic variants were not detected, but a variant of *STK11* (c.394T>C, p.C132R) was detected in the germline (as expected). The reference transcript used for annotation was ENST00000326873.12. The allele frequencies of the tumor and blood samples were 87.8% (1567/1795) and 42.0% (229/545), respectively. Importantly, this variant was registered as Likely Pathogenic in the NIH ClinVar public database (ID: 852152) and therefore confirmed the PJS diagnosis. We further performed upper gastrointestinal endoscopy and multiple polyps from stomach to duodenum were found (Figure 4a,b). However, pathological findings were mildly inflamed gastric mucosa with hyperplastic change (Figure 4c), inconsistent with typical PJS‐type polyps.

**FIGURE 5:**
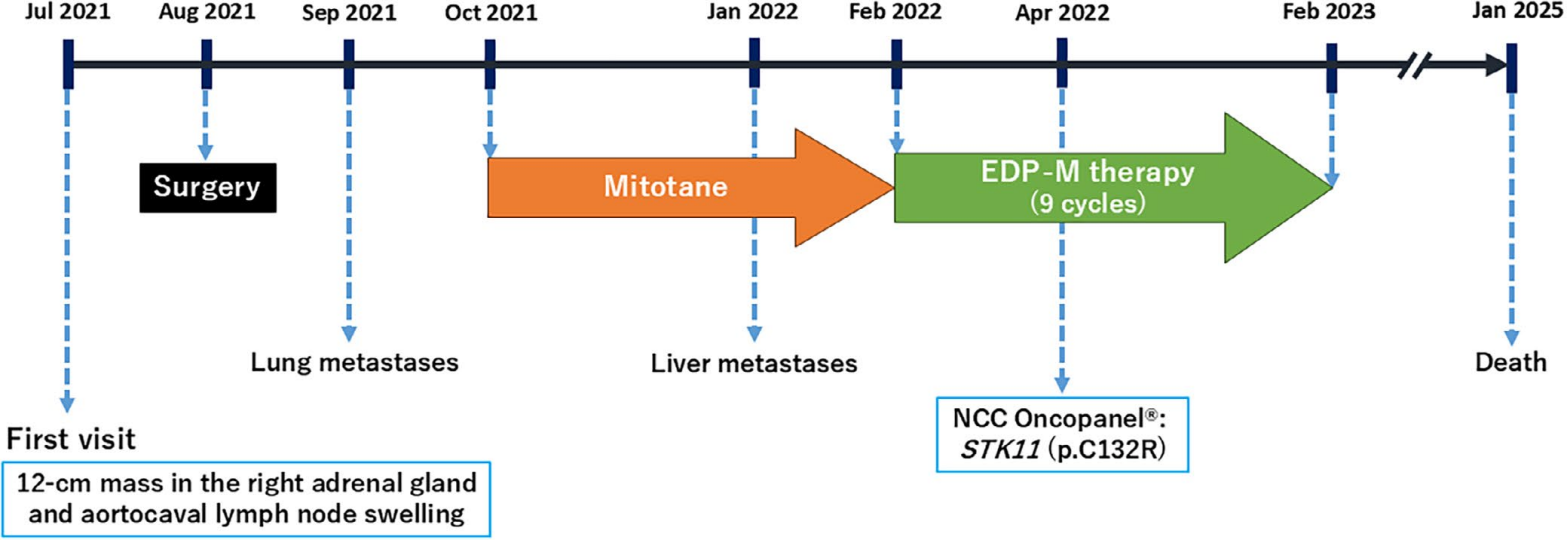
Timeline of the present case.

We performed a total of 9 cycles of EDP‐M therapy with treatment interruptions. The main adverse events were grade 4 neutropenia and grade 3 anemia, according to the Common Terminology Criteria for Adverse Events version 5.0 (CTCAE v5.0). The best observed response to EDP‐M therapy was stable disease (SD), according to the Response Evaluation Criteria in Solid Tumors (RECIST) 1.1. The patient received the best supportive care possible after 9 cycles of EDP‐M therapy for 1 year before achieving sustained SD for 1 year. Later, disease progression (PD) was confirmed, with death occurring 3 years after the start of EDP‐M therapy. A chronological summary of the case report is shown in Figure 5.

## 3 Discussion

PJS is an autosomal‐dominant genetic disease caused by a pathological variant of *STK11* and features gastrointestinal hamartomatous polyps, hyperpigmentation in mucocutaneous tissue, and increased epithelial malignancy risk. The prevalence is 1 in 50 000–200 000 individuals [1] and the most frequently associated malignancies are gastrointestinal tract (colon/rectum 39%, stomach 29%, small intestine 13%), followed by breast (54%) and pancreas (36%) [1]. However, ACC is only rarely complicated by PJS. To the best of our knowledge, there has been only one previous case reported by Yalçin et al., where a 16‐month‐old girl had PJS complicated by ACC and thyroid cancer [2]. This patient was diagnosed with ACC due to fever, vomiting, and weight loss, with a large left adrenal mass (10 × 12 cm) and a missense mutation in E199D.

*STK11* is a tumor suppressor encoding for a serine–threonine kinase that modulates cellular proliferation and polarity [2]. Guidelines for PJS state that pathological germline mutations in the *STK11* gene have been identified in 80%–94% of patients with PJS and one‐third have large deletions [1]. The present case had a germline variant of *STK11* (p.C132R) that was registered as Likely Pathogenic (only 1 submission) on ClinVar (as of February 2026) and as probably damaging in the PolyPhen‐2 analysis [5]. On the other hand, the gastric polyp was not pathologically diagnosed as hamartomatous, but a previous study revealed that stomach polyps were less frequently diagnosed as PJS‐type than small intestine polyps [6]. In fact, 8 of 11 PJS patients (72.7%) were diagnosed with hyperplastic polyps in the stomach, similar to the present case [6]. Therefore, to clinically confirm a diagnosis of PJS, we should have performed biopsies of polyps in the small intestine.

*STK11* germline mutations causative for PJS precipitate loss of function within the functional kinase domain [7]. Loss of *STK11* function then promotes mTOR signaling through inactivation of the AMPK/TSC pathway, leading to aberrant cell growth via metabolic reprogramming in cancer [7, 8]. However, *STK11* is not a hallmark tumor suppressor gene in ACC as Zheng et al. previously reported *TP53*, *ZNRF3*, *CTNNB1*, *PRKAR1A*, *CCNE1*, *TERF2*, *RPL2*, and *NF1* as driver genes for ACC [9]. Although *ZNRF3*, *PRKAR1A*, and *TERF2* are not examined by the OncoGuide NCC OncoPanel, no pathogenic variants of *TP53*, *CTNNB1*, *CCNE1*, or *NF1* were identified in this patient. On the other hand, preclinical studies reported mTOR inhibitor efficacy in ablating in vitro cellular proliferation in ACC cell lines and ACC xenograft growth in immunodeficient mice [3, 10, 11]. Importantly, the allele frequencies of the tumor and blood samples suggest loss of function of STK11 in the tumor. We speculate that pathogenic *STK11* variants might have contributed to ACC carcinogenesis or progression through mTOR activation in this patient; however, there is no definitive evidence establishing this relationship and further studies are required to elucidate the mechanism.

## 4 Conclusion

To our best knowledge, this is the second report of ACC associated with PJS in a rare germline variant of *STK11*. Although rare, loss of *STK11* function may lead to cancers outside expected sites in PJS patients.

## Author Contributions

**Satoshi Nitta:** writing – review and editing, data curation. **Tomoki Ishida:** writing – original draft, visualization, conceptualization. **Akio Hoshi:** writing – review and editing. **Shuya Kandori:** writing – review and editing, conceptualization, supervision, writing – original draft, methodology. **Reo Takahashi:** data curation, writing – review and editing. **Yoshiyuki Nagumo:** conceptualization, methodology, writing – review and editing. **Hiromitsu Negoro:** writing – review and editing. **Masanobu Shiga:** writing – review and editing, data curation. **Daisuke Matsubara:** writing – review and editing, data curation. **Hiroyuki Nishiyama:** writing – review and editing, supervision, conceptualization, methodology. **Shohei Sugita:** writing – review and editing, data curation. **Bryan J. Mathis:** writing – review and editing. **Takashi Kawahara:** writing – review and editing. **Takahito Nakajima:** writing – review and editing, data curation. **Toshitaka Ishiguro:** writing – review and editing, data curation.

## Funding

The authors have nothing to report.

## Ethics Statement

This case report was reviewed and approved by the University of Tsukuba Hospital Institutional Review Board (#R04‐150).

## Consent

Written informed consent for the publication of the case details and use of images was obtained from this patient.

## Conflicts of Interest

The authors declare no conflicts of interest.

## Data Availability

The data supporting the findings of this study are available from the corresponding author upon reasonable request.
